# Lymphadenectomy and risk of reoperation or mortality shortly after surgery for oesophageal cancer

**DOI:** 10.1038/srep36092

**Published:** 2016-10-31

**Authors:** Jesper Lagergren, Fredrik Mattsson, Andrew Davies, Mats Lindblad, Pernilla Lagergren

**Affiliations:** 1 Upper Gastrointestinal Surgery, Department of Molecular medicine and Surgery, Karolinska Institutet, Karolinska University Hospital, Stockholm, Sweden; 2 Division of Cancer Studies, King’s College London, Guy’s and St Thomas’ NHS Foundation Trust, London, United Kingdom; 3 Department of Clinical Science, Intervention and Technology, Karolinska Institutet, Stockholm, Sweden; 4 Surgical Care Sciences, Department of Molecular medicine and Surgery, Karolinska Institutet, Karolinska University Hospital, Stockholm, Sweden

## Abstract

The prognostic role of lymphadenectomy during surgery for oesophageal cancer is questioned. We aimed to test whether higher lymph node harvest increases the risk of early postoperative reoperation or mortality. A population-based cohort study including almost all patients who underwent resection for oesophageal cancer in Sweden in 1987–2010. Data were collected from medical records and well-established nationwide Swedish registries. The exposures were number of removed lymph nodes (primary) and number of node metastases (secondary). The main study outcome was reoperation/mortality within 30 days of primary surgery. Relative risks (RRs) with 95% confidence intervals (CIs) were calculated using Poisson regression, adjusted for age, sex, co-morbidity, neoadjuvant therapy, tumour stage, tumour histology, surgeon volume, and calendar period. Among 1,820 participants, the risk of reoperation/mortality did not increase with greater lymph node harvest (RR = 0.98, 95%CI 0.96–1.00, discrete variable) or with greater number of removed metastatic nodes (RR = 1.00, 95% CI 0.95–1.05, discrete variable). Similarly, in stratified analyses within pre-defined categories of tumor stage, surgeon volume and calendar period, increased number of removed nodes or node metastases did not increase the risk of reoperation/mortality. Lymphadenectomy during oesophageal cancer surgery is a safe procedure in the short term perspective.

Oesophageal cancer is one of the most common and deadly cancer types, and it is the 6^th^ most common cause of cancer death globally^1^. Surgical resection is the cornerstone of the curatively intended treatment of oesophageal cancer and survival following oesophagectomy has improved over the last few decades^2^. This improvement is likely due to multiple factors, including improvements in patient selection, perioperative care and centralisation of services. Yet, only 30% of patients survive 5 years following surgery according to recent population-based data^3^, which stresses the need to identify factors that can further optimise surgical treatment. The extent of lymph node removal might be a relevant factor in this respect. Although based on a limited body of evidence, the use of a fairly extensive (2-field) lymphadenectomy is generally recommended^4^,^5^,^6^. However, the independent prognostic role of lymphadenectomy has been questioned in two recent studies from our research group^7^,^8^. The possible lack of an independent association between lymph node harvest and long-term prognosis highlights the need to also examine the role of lymphadenectomy in relation to serious short-term outcomes, caused by postoperative complications. We hypothesized that the additional surgical trauma associated with a more extensive lymphadenectomy increases the risk of postoperative complications requiring reoperation, or leading to mortality within 30 days of oesophagectomy. To test this hypothesis, we conducted a population-based nationwide Swedish cohort study.

## Results

### Patients

The entire study cohort included 1 820 patients who underwent surgical resection for oesophageal cancer. Among these, 1 434 patients (79%) had complete data on all study exposures, covariates and outcomes, and were thus selected for the complete case analysis. Since the results were very similar for the entire cohort (imputation of missing data) and the cohort with all data available (complete case analysis), we present only the results for the entire cohort. Characteristics of the patients categorised into quartiles of number of lymph nodes are presented in Table 1. A greater number of removed nodes were associated with higher surgeon volume and more recent calendar period.

### Lymph node harvest and 30-day postoperative outcomes

An increased lymph node harvest did not increase the risk of reoperation/mortality within 30 days of surgery (Table 2). When the total number of lymph nodes was analysed as a discrete variable the RR was 0.98 (95% CI 0.96–1.00) and the corresponding RR for number of metastatic nodes was 1.00 (95% CI 0.95–1.05). Comparing the highest quartile of lymph node yield and node metastases with the lowest two quartiles produced RRs of 0.80 (95% CI 0.51–1.26) and 0.85 (95% CI 0.56–1.27), respectively. There were no statistically significant associations between number of lymph nodes or node metastases and any of the six outcomes when analysed separately (Table 2). In analyses stratified by categories of tumour stage, surgeon volume and calendar period, an increased lymph node harvest did not entail an increased risk of reoperation/mortality within 30 days of oesophagectomy (Table 3).

## Discussion

This study did not support the hypothesis of an increased risk of 30-day postoperative reoperation or mortality associated with an increased total number of lymph nodes or increased number of metastatic nodes removed.

Methodological advantages of the study include the population-based design with a high participation rate, robust assessment of exposures and outcomes, and the adjustment for several potential confounding factors. These factors counteract bias from selection, misclassification, and confounding, respectively. One can discuss whether reoperation is a good assessment of complications, but we deemed that the validity of this variable was substantially higher than evaluating individual complications that might easily have been missed in the discharge records, while reoperations are not. An obvious confounding factor in this study is surgeon volume, since surgeons with a higher annual volume of oesophagectomy also typically remove more lymph nodes and have better short-term outcomes than less experienced surgeons^7^,^9^. Therefore, we adjusted for annual surgeon volume in the multivariable model and also performed stratified analyses among surgeons above and below the median volume. Yet, we cannot entirely exclude residual confounding, e.g. due to rough categorization or misclassification. Another methodological issue is that reoperation and mortality are competing events, since mortality occurring before any potential later reoperation is not accounted for. Therefore, the combined reoperation/mortality outcome was selected as the main study outcome, while the results regarding the separate reoperation outcomes should be interpreted with caution. We used 30-day outcomes to avoid including reoperations for later complications that tend to be less severe, and the use of 30-day mortality rather than 90-day mortality was because this study focused on reoperations but included mortality because of the issue of competing events. Another source of error is missing data on some variables, including the lymph node variables. To account for this we used both imputation analysis (imputation where data were missing) and complete case analysis (including only patients with data on all variables). The similar results using both these analytical approaches indicate that the presence of missing data did not strongly influence the results of the study. Another source of error is misclassification of the assessment of the number of lymph nodes removed, which could differ between histopathologists. Such misclassification should be random, and thus dilute potential associations. However, our previous study on lymph node removal and long-term prognosis revealed strong associations between lymph node metastases and mortality, which lends validity to the node assessment^7^. Finally, despite the large sample size, the relatively low frequency of the study outcomes reduced the statistical precision, particularly in the subgroup analyses.

Two recent studies from our group indicate a lack of long-term prognostic benefit from a higher lymph node yield during surgery for oesophageal cancer after controlling for surgeon volume^7^,^8^. One was based on nationwide Swedish data^7^, and the other was based on data from a high-volume center for oesophageal cancer surgery in London, the United Kingdom^8^. On the other hand, the results from the present study indicate that a more extensive lymph node dissection might not be harmful. The lack of increased risk of serious poor short-term outcomes following a more extensive lymphadenectomy needs to be confirmed in future research before any clinical recommendations can be made. Although the volume of resected tissue can vary substantially between two surgeons, it is possible that standardization of the lymph node dissection after having conducted a substantial number of oesophagectomies makes this dissection quite safe. The issues following the additional surgical trauma associated with a more extensive lymphadenectomy might be counteracted by the increasing surgeon experience that tends to parallel the extent of lymph node removal.

In conclusion, this large and population-based cohort study provides no evidence that a more extensive lymphadenectomy increases the risk of reoperation or mortality within 30 days of primary resectional surgery for oesophageal cancer. Thus, lymphadenectomy can be regarded a safe procedure in the short term.

## Methods

### Design

The study was designed and analyzed according to a detailed study protocol which was developed and completed prior to the initiation of the study. Earlier versions of our nationwide Swedish cohort for oesophageal cancer surgery have been described in detail elsewhere^2^,^7^,^10^. In brief, the study cohort included virtually all patients (98%) who underwent open thoraco-abdominal resection for oesophageal cancer in Sweden during the period 1987 to 2010. The study exposures were the total number of lymph nodes removed and the number of lymph node metastases removed. Data on lymph node harvest were retrieved from the histopathology records of the resected tumour specimens throughout Sweden. The main study outcome was reoperation/mortality occurring within 30 days of primary oesophagectomy. Secondary outcomes were reoperation (for any reason), reoperation for anastomotic leakage, reoperation with laparotomy, reoperation for wound infection, and mortality, all within 30 days of the primary surgery. The data for assessing reoperation and mortality were collected from the Swedish Patient Registry (operation codes) and the Swedish Causes of Death Registry (date of death), respectively. Data on all relevant clinical variables as potential confounders (presented below) were retrieved from medical records, mainly histopathology records and operation charts.

### Approvals

The study and the study protocol were approved by the institutional and licensing committee, i.e. the Regional Ethical Review Board in Stockholm (reference number 12/537–32). All methods were carried out in accordance with relevant guidelines and regulations. Informed consent was not obtained since this is not required for this type of study (based on registry data and medical records) according to Swedish law.

### Statistical analysis

The number of lymph nodes and the number of lymph node metastases were analyzed both as continuous variables and categorical variables (quartiles) in relation to risk of the study outcomes. Since the number of nodes and node metastases was low in the first and second quartiles, these were collapsed into one group. Poisson regression was used to calculate relative risks (RRs) with 95% confidence intervals (CIs). Log-transformed person-time was included in the model as an offset. We assumed that each individual contributed the same weight towards person-time. RRs were adjusted for eight variables that were selected as potential confounders based on previous research: 1) age (continuous variable), 2) sex (categorized into male or female), 3) tumor stage (0-I, II or III-IV), 4) co-morbidity (Charlson co-morbidity index^11^: 0, 1 or >1), 5) neoadjuvant oncological therapy (yes or no), 6) histological tumour type (adenocarcinoma or squamous cell carcinoma), 7) annual surgeon volume of oesophagectomies (<17 or ≥17), and 8) calendar period (1987–1999 or 2000–2010). Furthermore, six more variables were created with a combination of either number of lymph nodes or the number of lymph node metastases and tumor stage (0-I, II and III-IV), annual surgeon volume of oesophagectomies (<17 and ≥17) and calendar period of surgery (1987–1999 and 2000–2010). These analyses were conducted to evaluate if the association between either number of lymph nodes or the number of lymph node metastases and the outcome reoperation/mortality might change depending on tumour stage, annual surgeon volume and calendar time. To handle missing data on exposures or covariates, we conducted both multiple imputation (using the entire cohort while imputing missing data) and complete case analysis (using only patients for whom all variables were available). Twenty data sets were imputed in the multiple imputation analysis. The monotone logistic method in PROC MI was used on the assumption that the missing data were missing at random (MAR)^12^. The variables included in the imputation were the eight potential confounders presented above, one of the exposures and one of the outcomes, depending on the model. Furthermore, PROC MIANALYZE was used to combine the results from the analyses of the 20 datasets. The underlying assumption was evaluated by means of sensitivity analyses utilizing pattern-mixture models using the statement ‘missing not at random’. The statistical software SAS version 9.4 (SAS Institute, Cary, NC) was used for all data management and statistical analysis.

## Additional Information

**How to cite this article**: Lagergren, J. *et al*. Lymphadenectomy and risk of reoperation or mortality shortly after surgery for oesophageal cancer. *Sci. Rep.* **6**, 36092; doi: 10.1038/srep36092 (2016).

**Publisher’s note:** Springer Nature remains neutral with regard to jurisdictional claims in published maps and institutional affiliations.

## Figures and Tables

**Table 1:** Characteristics of 1820 patients who underwent surgical resection for esophageal cancer in Sweden in 1987–2010, categorized for total number of lymph nodes removed.

		Number of lymph nodes in quartiles			
				1^st^/2^nd^ (0–7) Patients, N (%)	3^rd^ (8–15) Patients, N (%)	4^th^ (16–114) Patients, N (%)	Missing Patients, N (%)
Total		772 (100)	375 (100)	323 (100)	350 (100)
Mean age (standard deviation)		65 (10)	65 (9)	66 (9)	66 (10)
Sex	Male	578 (75)	284 (76)	246 (76)	252 (72)
	Female	194 (25)	91 (24)	77 (24)	98 (28)
Co-morbidity score	0	490 (63)	202 (54)	155 (48)	217 (62)
	1	144 (19)	84 (22)	77 (24)	70 (20)
	>1	138 (18)	89 (24)	91 (28)	63 (18)
Tumor stage	0-I	205 (27)	78 (21)	57 (18)	82 (23)
	II	297 (38)	120 (32)	116 (36)	129 (37)
	III-IV	270 (35)	176 (47)	149 (46)	127 (36)
	Missing	0 (0)	1 (0)	1 (0)	12 (3)
Tumor histology	Adenocarcinoma	290 (38)	194 (52)	172 (53)	136 (39)
	Squamous	482 (62)	181 (48)	151 (47)	210 (60)
	Missing	0 (0)	0 (0)	0 (0)	4 (1)
Neoadjuvant therapy	No	490 (63)	271 (72)	244 (76)	224 (64)
	Yes	282 (37)	104 (28)	79 (24)	124 (35)
	Missing	0 (0)	0 (0)	0 (0)	2 (1)
Surgeon volume	<17	427 (55)	158 (42)	99 (31)	203 (58)
	≥17	321 (42)	210 (56)	222 (69)	131 (37)
	Missing	24 (3)	7 (2)	2 (1)	16 (5)
Calendar period	1987–1999	540 (70)	120 (32)	22 (7)	310 (89)
	2000–2010	232 (30)	255 (68)	301 (93)	40 (11)
30-day reoperation/mortality		127 (16)	43 (11)	32 (10)	69 (20)
30-day mortality		49 (6)	10 (3)	7 (2)	32 (9)
30-day reoperation		91 (12)	36 (10)	26 (8)	47 (13)
Anastomotic leak		12 (2)	10 (3)	11 (3)	1 (0)
Laparotomy		25 (3)	10 (3)	7 (2)	15 (4)
Wound infection		24 (3)	3 (1)	1 (0)	14 (4)

**Table 2:** Lymph node harvest and risk of reoperation/mortality and other outcomes within 30 days of surgery for esophageal cancer (n = 1820), expressed as relative risk (RR) with 95% confidence interval (CI)^a^.

	Reoperation/mortality (271 patients)	Mortality (98 patients)	Reoperation (200 patients)	Anastomotic leak (34 patients)	Laparotomy (57 patients)	Wound infection (42 patients)
	RR (95% CI)	RR (95% CI)	RR (95% CI)	RR (95% CI)	RR (95% CI)	RR (95% CI)
**Number of nodes**						
Discrete variable (0–114)^b^	0.98 (0.96–1.00)	0.97 (0.93–1.01)	0.98 (0.96–1.01)	1.01 (0.98–1.04)	1.00 (0.96–1.04)	0.96 (0.88–1.04)
Quartile 1^st^ and 2^nd^ (0–7)	1 (reference)	1 (reference)	1 (reference)	1 (reference)	1 (reference)	1 (reference)
Quartile 3^rd^ (8–15)	0.84 (0.59–1.21)	0.60 (0.31–1.18)	0.90 (0.60–1.36)	1.39 (0.57–3.44)	1.26 (0.58–2.74)	0.54 (0.17–1.74)
Quartile 4^th^ (16–114)	0.80 (0.51–1.26)	0.63 (0.26–1.55)	0.79 (0.47–1.33)	1.39 (0.54–3.62)	1.28 (0.48–3.38)	0.48 (0.06–3.64)
**Number of metastatic nodes**						
Discrete variable (0–44)^b^	1.00 (0.95–1.05)	1.00 (0.92–1.09)	1.00 (0.94–1.05)	1.08 (1.02–1.15)	1.04 (0.95–1.13)	0.99 (0.86–1.14)
Quartile 1^st^ and 2^nd^ (0–1)	1 (reference)	1 (reference)	1 (reference)	1 (reference)	1 (reference)	1 (reference)
Quartile 3^rd^ (2–3)	1.04 (0.69–1.57)	1.14 (0.56–2.32)	1.03 (0.65–1.65)	0.62 (0.18–2.21)	1.07 (0.41–2.77)	1.24 (0.43–3.57)
Quartile 4^th^ (4–44)	0.85 (0.56–1.27)	0.95 (0.50–1.82)	0.78 (0.48–1.26)	0.90 (0.32–2.59)	1.07 (0.45–2.55)	1.02 (0.37–2.83)

^a^Adjusted for age, sex, tumor stage, Charlson co-morbidity score, neoadjuvant therapy, tumor histology, surgeon volume, and calendar period.

^b^Included in the model as a discrete variable to evaluate linear trend.

**Table 3:** Lymph node harvest and metastasis and risk of reoperation/mortality within 30 days of surgery for esophageal cancer (n = 1820), stratified by tumor stage, surgeon volume and calendar period, expressed as relative risk (RR) with 95% confidence interval (CI)*.

Reoperation/mortality						
	Number of nodes			Number of metastatic nodes		
	Quartile			Quartile		
	1^st^ and 2^nd^ (0–7)	3^rd^ (8–15)	4^th^ (16–114)	1^st^ and 2^nd^ (0–1)	3^rd^ (2–3)	4^th^ (4–44)
	RR (95% CI)	RR (95% CI)	RR (95% CI)	RR (95% CI)	RR (95% CI)	RR (95% CI)
**Tumor stage**						
0-I	1 (reference)	0.82 (0.38–1.80)	0.63 (0.22–1.81)	1 (reference)	1.23 (0.17–9.04)	Not applicable
II	1.12 (0.75–1.66)	1.06 (0.60–1.88)	0.88 (0.45–1.71)	1.16 (0.82–1.63)	1.34 (0.70–2.58)	0.83 (0.36–1.93)
III-IV	1.03 (0.68–1.57)	0.79 (0.45–1.36)	0.90 (0.48–1.68)	1.07 (0.68–1.67)	1.05 (0.62–1.78)	0.95 (0.61–1.47)
**Surgeon volume**						
<17	1 (reference)	0.85 (0.52–1.39)	0.73 (0.38–1.41)	1 (reference)	0.94 (0.54–1.64)	0.90 (0.56–1.46)
≥17	0.74 (0.54–1.03)	0.62 (0.39–0.98)	0.63 (0.38–1.06)	0.74 (0.55–1.00)	0.87 (0.49–1.52)	0.56 (0.32–1.00)
**Calendar period**						
1987–1999	1 (reference)	0.96 (0.61–1.51)	0.79 (0.28–2.21)	1 (reference)	1.01 (0.59–1.73)	0.91 (0.56–1.46)
2000–2010	0.79 (0.53–1.18)	0.53 (0.34–0.84)	0.58 (0.38–0.88)	0.66 (0.47–0.92)	0.70 (0.39–1.24)	0.49 (0.27–0.88)

^*^Adjusted for age, sex, tumor stage, Charlson co-morbidity score, neoadjuvant therapy, tumor histology, surgeon volume, and calendar period.

## References

[b1] Torre L. A. . Global cancer statistics, 2012. *CA: a cancer journal for clinicians* 65, 87–108, 10.3322/caac.21262 (2015). 25651787

[b2] Rouvelas I. . Survival after surgery for oesophageal cancer: a population-based study. The lancet oncology 6, 864–870 (2005). 16257794 10.1016/S1470-2045(05)70347-8

[b3] Rutegard M. . Population-based esophageal cancer survival after resection without neoadjuvant therapy: an update. Surgery 152, 903–910, 10.1016/j.surg.2012.03.025 (2012). 22657730

[b4] Allum W. H. . Guidelines for the management of oesophageal and gastric cancer. Gut 60, 1449–1472, 10.1136/gut.2010.228254 (2011). 21705456

[b5] Lagarde S. M., Vrouenraets B. C., Stassen L. P. & van Lanschot J. J. Evidence-based surgical treatment of esophageal cancer: overview of high-quality studies. The Annals of thoracic surgery 89, 1319–1326, 10.1016/j.athoracsur.2009.09.062 (2010). 20338377

[b6] Rizk N. P. . Optimum lymphadenectomy for esophageal cancer. Annals of surgery 251, 46–50, 10.1097/SLA.0b013e3181b2f6ee (2010). 20032718

[b7] van der Schaaf M., Johar A., Wijnhoven B., Lagergren P. & Lagergren J. Extent of lymph node removal during esophageal cancer surgery and survival. Journal of the National Cancer Institute 107, 10.1093/jnci/djv043 (2015). 25748792

[b8] Lagergren J. . Extent of Lymphadenectomy and Prognosis After Esophageal Cancer Surgery. JAMA surgery, 1–8, 10.1001/jamasurg.2015.2611 (2015). 26331431

[b9] Birkmeyer J. D. . Surgeon volume and operative mortality in the United States. The New England journal of medicine 349, 2117–2127, 10.1056/NEJMsa035205 (2003). 14645640

[b10] Derogar M., Sadr-Azodi O., Johar A., Lagergren P. & Lagergren J. Hospital and surgeon volume in relation to survival after esophageal cancer surgery in a population-based study. J Clin Oncol 31, 551–557, 10.1200/JCO.2012.46.1517 (2013). 23295792

[b11] Charlson M. E., Pompei P., Ales K. L. & MacKenzie C. R. A new method of classifying prognostic comorbidity in longitudinal studies: development and validation. Journal of chronic diseases 40, 373–383 (1987). 3558716 10.1016/0021-9681(87)90171-8

[b12] Little R. & Rubin D. Statistical analysis with missing data. Wiley-Interscience (2002).

